# Effectiveness of Gyejibongnyeong-Hwan for shoulder pain: study protocol for a randomised, wait-list controlled pilot trial

**DOI:** 10.1186/s13063-020-4092-y

**Published:** 2020-02-17

**Authors:** Soobin Jang, Hyung Kyung Sung, Mi Mi Ko, Seon Mi Shin, Ho-Yeon Go, Jeeyoun Jung

**Affiliations:** 1 Clinical Medicine Division, Korea Institute of Oriental Medicine, Daejeon, Republic of Korea; 2 Department of Pediatrics, College of Korean Medicine, Semyung University, Jecheon, Republic of Korea; 3 Clinical Research Coordinating Team, Korea Institute of Oriental Medicine, Daejeon, Republic of Korea; 4 Internal Medicine, College of Korean Medicine, Semyung University, Jecheon, Republic of Korea

**Keywords:** Gyejibongnyeong-Hwan, Gui-Zhi-Fu-Ling-Wan, Keishibukuryogan, Shoulder pain, Hyperlipidaemia, Blood stasis

## Abstract

**Background:**

Shoulder pain is an uncomfortable feeling in the muscle around the shoulder. The cause of myalgia is the accumulation of lactic acid in muscles and impaired blood circulation, which is called blood stasis in traditional East Asian medicine. This study aimed to explore the therapeutic effect of Gyejibongnyeong-Hwan (GBH) for shoulder discomfort related to blood stasis before and after treatment.

**Methods/design:**

This study will be a double-centre, randomised, wait-list controlled pilot trial. Participants with shoulder pain and with a visual analogue scale score of 4 or higher out of 10, blood stasis score of 9 or higher, and triglyceride level of ≥150 mg/dl or total cholesterol level of ≥200 mg/dl will be recruited from two university hospitals. A total of 40 participants will be assigned to the immediate and waiting treatment groups. The immediate treatment group will receive GBH for 8 weeks on enrolment while the waiting treatment group will receive GBH for 8–16 weeks after 8 weeks of controlled waiting. The primary outcome is shoulder pain, and the secondary outcomes are the blood stasis score, blood pressure, ankle–brachial pressure index, brachial–ankle pulse wave velocity, body mass index, waist circumference, indexes of oximetry, and levels of blood lipid, blood sugar, resistin, C-reactive protein, serum amyloid P, and D-dimer.

**Discussion:**

The results of this pilot trial will be the bases for a full-scale clinical trial of GBH.

**Trial registration:**

Clinical Research Information Service, KCT0003837. Registered on 23 April 2019. https://cris.nih.go.kr/cris/en/search/search_result_st01.jsp?seq=14258

## Background

Shoulder pain is a feeling of stiffness or discomfort around the shoulder joint, especially in the trapezius muscle. The incidence of shoulder pain in primary care is 14.7 per 1000 patients per year, and approximately 70% of the population experiences more than one episode in a lifetime [1–3]. Electrotherapy (transcutaneous electrical nerve stimulation, TENS), ultraviolet therapy, hot pack, massage, and sodium channel blocker drugs are used to alleviate muscle contractions; however, these interventions are largely ineffective [4, 5]. The tension in the shoulder muscles worsens with prolonged sitting at a computer and improper posture, and under physical and emotional stress. Myalgia is caused by the accumulation of lactic acid in the muscles and impaired blood circulation. In traditional East Asian medicine, muscle cramps and chronic musculoskeletal pain are considered to be due to blood stasis and can lead to limitations in shoulder movements by blocking the meridians [6].

Blood stasis is defined as the pathological stagnation of blood in certain parts of the body. It manifests as a variety of symptoms, including pain in a fixed location, a dark-purple appearance of the face or tongue, bleeding, blood spots under the skin, and an astringent pulse [7]. It is believed that blood stasis causes lipoprotein disorders such as thrombosis and the formation of atheromatous plaques [8–10]. Several herbal formulae that reduce blood stasis are helpful in the management of metabolic disorders [11]. Furthermore, blood stasis can occur after physical injuries, and the use of herbal medicine to eliminate blood stasis has been reported to alleviate muscle pain in some cases [12].

Therefore, we hypothesised that Gyejibongnyeong-Hwan (GBH), a herbal formula for dissipating blood stasis, is effective for the treatment of shoulder pain and hyperlipidaemia. GBH was approved as a medicine for shoulder discomfort by the Ministry of Food and Drug Safety (MFDS), South Korea [13]. In previous studies, GBH has been reported to ameliorate metabolic diseases such as hyperglycaemia and hyperlipidaemia [14, 15]. The primary objective of this pilot trial is to explore whether GBH relieves shoulder pain related to blood stasis. The secondary objective is to determine whether GBH is also helpful in improving blood lipid levels associated with blood stasis.

## Methods

### Study design

**Fig 1:**
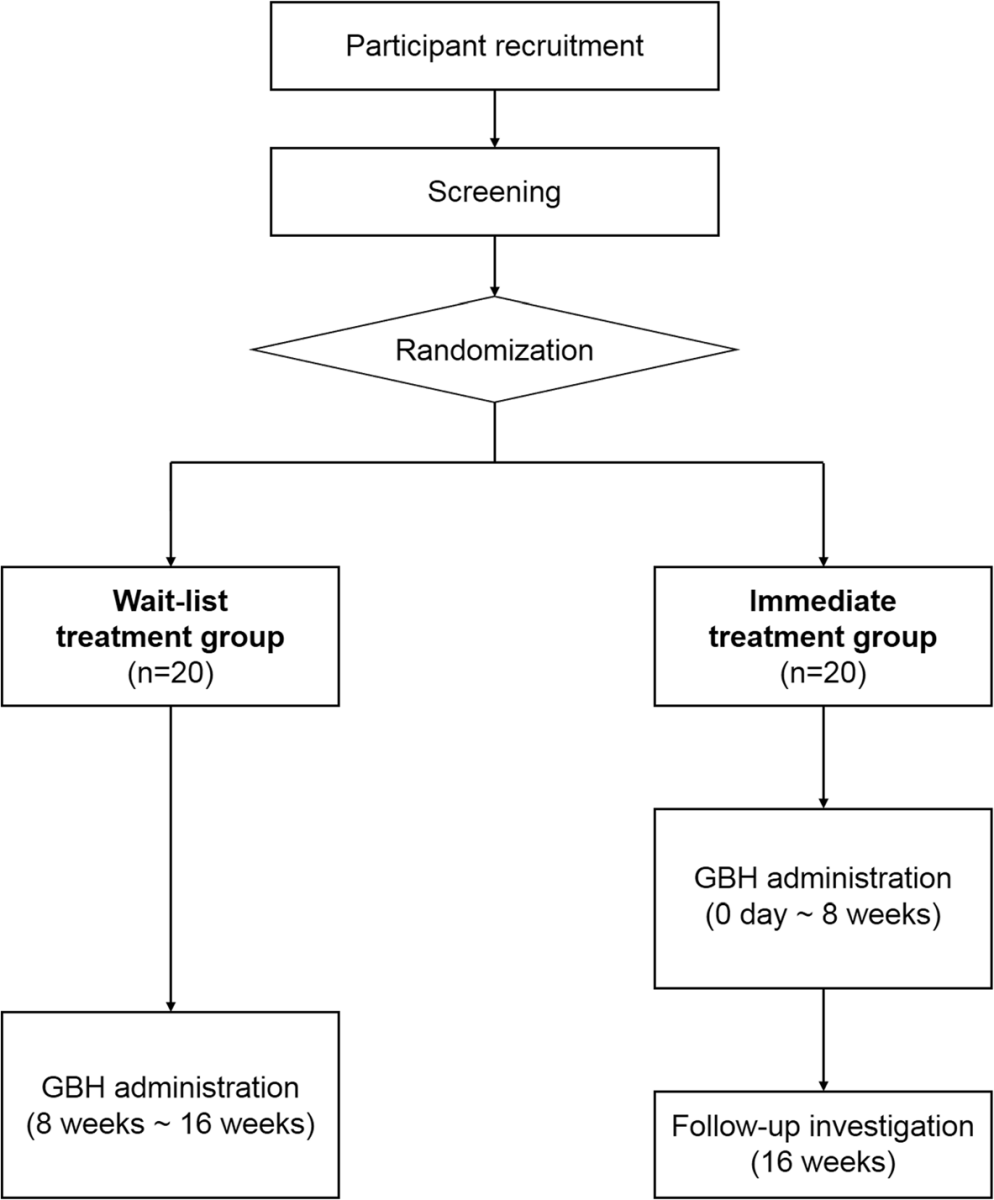
Flow chart of the trial process. GBH Gyejibongnyeong-Hwan

This is a multi-centre, randomised, wait-list controlled pilot study. This study will determine the effectiveness of GBH by comparing two groups: the immediate GBH treatment group and the waiting treatment group. Furthermore, the findings before and after GBH treatment will be compared in each group. The immediate treatment group will undergo an 8-week treatment period and an 8-week follow-up period. The waiting treatment group will undergo an 8-week waiting period and an 8-week treatment period. An overview of the trial process is shown in Fig. 1.

### Setting

This trial will be conducted at two hospitals: Chungju Oriental Hospital of Semyung University and Jecheon Oriental Hospital of Semyung University. This study was sponsored by Korea Institute of Oriental Medicine (KIOM), and KIOM wrote the protocol following the Standard Protocol Items: Recommendations for Interventional Trials (SPIRIT) checklist (Additional file 1). Both centres will be managed by KIOM during the study period.

### Recruitment

Patients with shoulder pain and blood stasis will be recruited over 6 months at Chungju Oriental Hospital of Semyung University and Jecheon Oriental Hospital of Semyung University. Posters will be posted publicly inside and outside both hospitals. Anyone interested in the study can contact the clinical research coordinator (CRC) of each site via telephone or e-mail. Then, a Korean medical doctor will screen participants for eligibility. Those who agree to participate in the study and provide written informed consent will be enrolled.

### Inclusion and exclusion criteria

The following participants will be included in this trial: those having physical strength, cold feet, and shoulder pain and a visual analogue scale (VAS) score of 4 or higher out of 10; those with a blood stasis score of 9 or higher (15-item questionnaire for diagnosis of blood stasis with metabolic diseases) (Appendix: Table 2); triglyceride (TG) level of ≥150 mg/dl or total cholesterol (TC) level of ≥200 mg/dl; and those who voluntarily agree to participate in this study and will comply with the restriction during the study period.

The following patients were excluded: those who are unable to give informed consent by themselves; pregnant women (for women of child-bearing age, the human chorionic gonadotropin (HCG) level will also be measured before GBH administration); those participating in another clinical trial; and those receiving medicines for therapeutic purposes.

### Randomisation, allocation, and blinding

Participants meeting the inclusion and exclusion criteria will be randomly allocated to either the immediate treatment group or the waiting treatment group at a ratio of 1:1. A computer-generated randomisation schedule based on a table of random digits will be used for group assignment. Access to the sequence will be restricted to the KIOM data manager (who is independent of the study team). The randomisation sequence will be included in a password-protected computer file and allocated to each hospital by an investigator.

Since this is an open-label trial without placebo, neither researchers nor participants will be blinded.

### Intervention

The immediate treatment group will receive GBH for 8 weeks on enrolment, and the waiting treatment group will receive GBH for 8–16 weeks at 8 weeks after enrolment. Every participant will receive 3.0 g of GBH (Hanpoong Pharm & Food Co., Ltd) twice a day for 8 weeks. GBH is a light-brown granular substance consisting of Cinnamomi Ramulus, *Wolfiporia extensa*, *Paeonia suffruticosa* Andrews, *Prunus persica*, and *Paeonia lactiflora* in an equal ratio. Any other medicine or treatment that may affect shoulder pain and hyperlipidaemia will be prohibited during the study period.

### Sample size

This trial is a pilot study that examines the feasibility of conducting a large-scale randomised clinical trial for the use of GBH to treat shoulder pain related to blood stasis. No adequate references could be found with regard to the sample size; hence, a sample size of 40 patients is determined to be adequate considering a 25% drop-out rate. Participants will be assigned to either the immediate treatment group or the waiting treatment group at a ratio of 1:1.

### Data collection

The following demographic data will be collected on the enrolment date: age, sex, occupation, height, weight, alcohol habit, and smoking status. Blood tests and blood stasis questionnaire evaluation will be conducted and VAS scores for shoulder pain will be assessed during the screening visit. After randomisation, the ankle–brachial pressure index (ABI), brachial–ankle pulse wave velocity (baPWV), oxygen saturation (SpO_2_), total haemoglobin (SpHb), pleth variability index (PVI), oxygen content (SpOC), pulse rate (PR), methaemoglobin (SpMet), perfusion index (PI), blood pressure (BP), body mass index (BMI), and waist circumference (WC) will be assessed (visit 1). Blood tests, blood stasis questionnaire evaluation, and VAS score evaluation will be conducted again during visit 1 in the waiting treatment group since the subjects are required to wait for 8 weeks after randomisation. SpO_2_, SpHb, PVI, SpOC, PR, SpMet, and PI will be measured using an oximeter (Masimo Corp.). Blood tests, blood stasis questionnaire evaluation, and oximeter measurements will be performed and shoulder pain, ABI, baPWV, BP, BMI, and WC will be assessed every 4 weeks after GBH has been received (visits 2 and 3). Furthermore, compliance and adverse events will be assessed every 4 weeks (visit 2 and 3), and patient satisfaction will be evaluated after 8 weeks of intervention (visit 3). At the follow-up visit in the immediate treatment group, blood tests, blood stasis questionnaire evaluation, oximeter measurements, assessment of shoulder pain, and determination of ABI, baPWV, BP, BMI, and WC will be performed (visit 4). The time points of the evaluations are presented in Table 1.

**Table 1:**
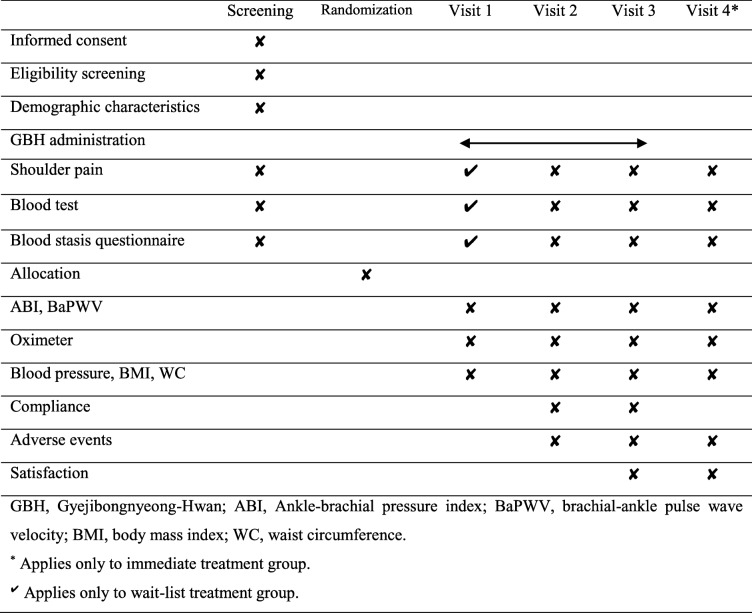
Study schedule of the GBH pilot study

*ABI* ankle–brachial pressure index, *BaPWV* brachial–ankle pulse wave velocity, *BMI* body mass index, *GBH* Gyejibongnyeong-Hwan, *WC* waist circumference

^*^ Applies only to immediate treatment group

✔ applies only to wait-list treatment group

### Outcome measures

#### Primary outcome

The primary outcome is shoulder pain. This will be assessed using VAS scores, which are the patient-reported outcome. Lower VAS scores will indicate that GBH successfully reduced shoulder pain.

#### Secondary outcomes

The secondary outcomes include the blood stasis score, the high-density lipoprotein cholesterol (HDL-C), low-density lipoprotein cholesterol (LDL-C), TG, TC, fasting blood glucose (FBG), resistin, C-reactive protein (CRP), serum amyloid P (SAP), and D-dimer levels, ABI, baPWV, BP, BMI, WC, and the seven oximetry indices (SpO_2_, SpHb, PVI, SpOC, PR, SpMet, and PI). Blood stasis scores, ABI, baPWV, oximetry indexes, and levels of resistin, CRP, SAP, and D-dimer will be used to evaluate blood stasis. CRP, as an inflammation marker, will be also used as an outcome of trapezius myalgia treatment [16]. HDL-C, LDL-C, TG, TC, and FBG levels, BP, BMI, and WC will be measured to verify the effectiveness of GBH for treating metabolic diseases referred to in previous studies.

### Statistical analyses

All analyses will be conducted according to the intention-to-treat (ITT) principles. All statistical analyses will be two-sided, and statistical significance will be set at 0.05. The last-observation-carried-forward (LOCF) method will be used to input missing values. An independent *t* test or Wilcoxon rank-sum test will be used to compare changes in scores after performing the Kolmogorov–Smirnov test to confirm the normality of the distribution. All values will be presented as mean and standard deviation values or *n* (%) unless stated otherwise. Outcome variables will be analysed using the corresponding analysis of covariance (ANCOVA) considering the baseline scores as covariates. All statistical analyses will be performed using the SAS statistical package, version 9.4 (SAS Inc., Cary, NC, USA).

### Data management and monitoring

All data will be recorded electronically by the CRCs. All study-related data will be stored on secure servers at KIOM with frequent back-up procedures in place. Data quality will be ensured by conducting automatic validity and range checks during data entry. Both the principal investigators and the KIOM research team can access the final data sets, and all data will be password protected. The independent data monitoring team of KIOM whose members do not participate in this study will be the data monitoring committee. The trial institutions will be monitored by clinical research associates (CRAs) from KIOM while the study is ongoing; the CRAs will be independent of the research team. The CRAs will conduct monitoring visits at each site at least every 2 months after the enrolment of the first participant. They will review the source documents to confirm whether the data reported in the web-based system are complete and accurate.

### Safety and adverse events

All adverse events due to GBH will be reported in detail and participants showing any adverse event will be treated appropriately by doctors. The most common adverse reactions are expected to be skin rash, itching, anorexia, and diarrhoea. Adverse reactions will be reported to the institutional review board (IRB), and serious and unexpected adverse reactions will be reported to the regulatory authorities.

## Discussion

The aim of this study, as a pilot trial, is to observe the clinical effectiveness of GBH. The study treatment GBH (known as Gyejibongnyeong-Hwan in Korean medicine, Gui-Zhi-Fu-Ling-Wan in traditional Chinese medicine, and Keishibukuryogan in Kampo medicine) is a herbal medicine used to eliminate blood stasis. It consists of five herbs (Cinnamomi ramulus, *W. extensa*, *P. suffruticosa* Andrews, *P. persica*, and *P. lactiflora*) [17, 18]. GBH has been used mainly for treating primary dysmenorrhoea, uterine myoma [19], and climacteric symptoms such as hot flashes [18]. A phase II trial to assess the efficacy of GBH (TU-025) Tsumura & Co (Tokyo, Japan) for hot flash management was conducted in the United States; however, the results did not show significant clinical effectiveness. GBH has also been reported to be effective in the management of metabolic diseases [14]. In South Korea, GBH is one of the most popular herbal medicines for blood stasis management and has been approved as a treatment for shoulder discomfort by the MFDS [13]. Since GBH has not yet been approved for hyperlipidaemia, indices related to blood lipids will be determined as secondary outcomes. Our research team has plans for further clinical trials for Investigational New Drug application, depending on the results of this pilot trial.

The outcomes used to evaluate blood stasis were also designated as study variables for this pilot trial. A blood stasis questionnaire was developed by KIOM, the results of which will be evaluated based on standard guidelines by Korean medical doctors [20]. A questionnaire for blood stasis with metabolic diseases was developed and published recently [21]. This is an abbreviated version of the original questionnaire; the new questionnaire comprises 15 questions (from the original 31 questions) related to metabolic diseases. The 15-item questionnaire will be used at screening, and after registration, blood stasis will be evaluated using the original questionnaire (Appendix: Table 3). Outcomes associated with oximeter measurements and some biomarkers (i.e., resistin, CRP, SAP, and D-dimer) have been used in previous blood stasis studies and will also be measured in this pilot trial to determine whether they are associated with blood stasis. The ABI and baPWV indicate narrowing and stiffening of arteries and are therefore expected to be appropriate for the evaluation of blood stasis [22].

There are some limitations to this pilot study. First, this is an open-label, wait-list controlled trial in which neither the participants nor the clinical practitioners will be blinded. We could have designed a randomised controlled trial using a placebo; however, we decided to administer GBH to both groups. Since the sample size is relatively small, we adopted a wait-list controlled design that would allow the evaluation of GBH effectiveness, without reducing the number of participants receiving GBH. Second, the outcome measures for shoulder pain are only the VAS score and the CRP level, and these may not be enough. However, this study has been designed to minimise the burden of participants and the number of patient-reported outcomes. Furthermore, the blood stasis questionnaire contains pain-related items; therefore, other measurements for shoulder pain were not included in this study, such as the Shoulder Pain and Disability Index (SPADI) and the Disabilities of the Arm, Shoulder and Hand (DASH) questionnaire [23]. Third, a follow-up investigation will not be performed in the waiting group, due to the length of the entire study period and research funds. Fourth, the VAS score, which is the primary outcome, is self-reported, and there may be a measurement bias. Nonetheless, this study is meaningful because the findings of this study will serve as a reference for designing the full-scale randomised controlled trial.

### Trial status

This document is based on version 1.5 (May 31, 2019) of the study protocol. Recruitment started on June 3, 2019, and is expected to complete by March 31, 2020. As of July 2019, five participants had been enrolled in this study and no one has yet completed the study. The trial is ongoing.

### Supplementary information

**Additional file 1.** SPIRIT 2013 Checklist: Recommended items to address in a clinical trial protocol and related documents.

## Data Availability

Not applicable.

## Appendix

**Table 2:** Original version of the blood stasis questionnaire

Question	None	Mild	Moderate	Severe	Very severe
1. Having dizziness	①	②	③	④	⑤
2. Having central-type palsy	①	②	③	④	⑤
3. Sublingual varicosities	①	②	③	④	⑤
4. Dark purple of palate mucosa	①	②	③	④	⑤
5. Blackish red gingiva	①	②	③	④	⑤
6. Blackish red lips	①	②	③	④	⑤
7. Blackish red tongue	①	②	③	④	⑤
8. Ecchymosis of tongue	①	②	③	④	⑤
9. Dark colouration of periocular region	①	②	③	④	⑤
10. A dark colouration of the face	①	②	③	④	⑤
11. Having chest pain without angina pectoris	①	②	③	④	⑤
12. Side pain	①	②	③	④	⑤
13. Tenderness and resistance of hypochondrium	①	②	③	④	⑤
14. Tenderness and resistance of navel region	①	②	③	④	⑤
15. Lower abdominal pain	①	②	③	④	⑤
16. Tenderness and resistance of ileocecum	①	②	③	④	⑤
17. Tenderness and resistance of sigmoid colon	①	②	③	④	⑤
18. Haemorrhoid	①	②	③	④	⑤
19. Dark stool	①	②	③	④	⑤
20. Having pain due to sprain of ankle, wrist, and spine	①	②	③	④	⑤
21. Having symptoms (pain, bruise) due to contusion or traffic accident	①	②	③	④	⑤
22. Chronic pain in joint/palsies and numbness	①	②	③	④	⑤
23. Stabbing pain	①	②	③	④	⑤
24. Pain at night	①	②	③	④	⑤
25. Ecchymosis of skin	①	②	③	④	⑤
26. Scaly and rough skin	①	②	③	④	⑤
27. Telangiectasia or angioectasia	①	②	③	④	⑤
28. Palmar erythema	①	②	③	④	⑤
29. Bruised easily	①	②	③	④	⑤
30. Angina pectoris	① No		② Yes		
31. Rough pulse	① No		② Yes		

**Table 3:** 15-item questionnaire for the diagnosis of blood stasis with metabolic diseases

Question	Response		Score
1. Angina pectoris	No	Yes	3 points
2. Having chest pain without angina pectoris	No	Yes	
3. Blackish red tongue	No	Yes	
4. Ecchymosis of tongue	No	Yes	
5. Stabbing pain	No	Yes	
6. Sublingual varicosities	No	Yes	2 points
7. Dark purple of palate mucosa	No	Yes	
8. Blackish red lips	No	Yes	
9. Blackish red gingiva	No	Yes	
10. Chronic pain in joint/palsies and numbness	No	Yes	
11. Pain at night	No	Yes	
12. Bruised easily	No	Yes	
13. Dark colouration of periocular region	No	Yes	1 point
14. A dark colouration of the face	No	Yes	
15. Scaly and rough skin	No	Yes	

## Abbreviations

ABI: Ankle–brachial pressure index
ANCOVA: Analysis of covariance
baPWV: Brachial–ankle pulse wave velocity
BMI: Body mass index
BP: Blood pressure
CRIS: Clinical Research Information Service
CRP: C-reactive protein
FBG: Fasting blood glucose
GBH: Gyejibongnyeong-Hwan
HCG: Human chorionic gonadotropin
HDL-C: High-density lipoprotein cholesterol
ITT: Intention to treat
KIOM: Korea Institute of Oriental Medicine
LDL-C: Low-density lipoprotein cholesterol
LOCF: Last observation carried forward
MFDS: Ministry of Food and Drug Safety
PI: Perfusion index
PR: Pulse rate
PVI: Pleth variability index
SAP: Serum amyloid P
SpHb: Total haemoglobin
SpMet: Methaemoglobin
SpO_2_: Oxygen saturation
SpOC: Oxygen content
TC: Total cholesterol
TG: Triglyceride
VAS: Visual analogue scale
WC: Waist circumference
