# Correction to: Effectiveness of Gyejibongnyeong-Hwan for shoulder pain: study protocol for a randomised, wait-list controlled pilot trial

**DOI:** 10.1186/s13063-020-4210-x

**Published:** 2020-03-06

**Authors:** Soobin Jang, Hyun Kyung Sung, Mi Mi Ko, Seon Mi Shin, Ho-Yeon Go, Jeeyoun Jung

**Affiliations:** 1 Clinical Medicine Division, Korea Institute of Oriental Medicine, Daejeon, Republic of Korea; 2 Department of Pediatrics, College of Korean Medicine, Semyung University, Jecheon, Republic of Korea; 3 Clinical Research Coordinating Team, Korea Institute of Oriental Medicine, Daejeon, Republic of Korea; 4 Internal Medicine, College of Korean Medicine, Semyung University, Jecheon, Republic of Korea

**Correction to: Trials**

**https://doi.org/10.1186/s13063-020-4092-y**

After publication of our article [1] the authors have notified us that one of the names has been incorrectly spelled.

- Original name spelling:
  Hyung Kyung Sung
- Correct name spelling:
  Hyun Kyung Sung

The original article has been corrected.
